# From Grey Scale B-Mode to Elastosonography: Multimodal Ultrasound Imaging in Meningioma Surgery—Pictorial Essay and Literature Review

**DOI:** 10.1155/2015/925729

**Published:** 2015-05-25

**Authors:** Francesco Prada, Massimiliano Del Bene, Alessandro Moiraghi, Cecilia Casali, Federico Giuseppe Legnani, Andrea Saladino, Alessandro Perin, Ignazio Gaspare Vetrano, Luca Mattei, Carla Richetta, Marco Saini, Francesco DiMeco

**Affiliations:** ^1^Department of Neurosurgery, IRCCS Foundation Neurological Institute “C. Besta”, 20133 Milan, Italy; ^2^University of Milan, 20122 Milan, Italy; ^3^Department of Neurosurgery, Johns Hopkins University, Baltimore, MD 21218, USA

## Abstract

The main goal in meningioma surgery is to achieve complete tumor removal, when possible, while improving or preserving patient neurological functions. Intraoperative imaging guidance is one fundamental tool for such achievement. In this regard, intra-operative ultrasound (ioUS) is a reliable solution to obtain real-time information during surgery and it has been applied in many different aspect of neurosurgery. In the last years, different ioUS modalities have been described: B-mode, Fusion Imaging with pre-operative acquired MRI, Doppler, contrast enhanced ultrasound (CEUS), and elastosonography. In this paper, we present our US based multimodal approach in meningioma surgery. We describe all the most relevant ioUS modalities and their intraoperative application to obtain precise and specific information regarding the lesion for a tailored approach in meningioma surgery. For each modality, we perform a review of the literature accompanied by a pictorial essay based on our routinely use of ioUS for meningioma resection.

## 1. Introduction

Main goal of meningioma surgery is to obtain the complete tumor resection in order to reduce the recurrence rate but preserve or improve the patient's neurological functions [1, 2]. In many cases, this is a difficult achievement, because of the risk of damages to arteries, sinuses, cranial nerves or other neighbors relevant structures. Surgical morbidity and mortality are mainly related to tumor location and volume [3].

*Image Guided Surgery*. It represents the gold standard technique in order to correctly perform the surgical planning, facilitate tumor removal, identify relevant neurovascular structures, and maximize the safety and degree of excision [4, 5].

The more commonly available and routinely used tools for intraoperative image guidance are the neuronavigation systems (NNs), which are based on preoperative imaging. NN is an excellent tool for surgical planning and identification of the lesion and the surrounding vital structures but suffers from major limitations. Being based on preoperative acquired images, it does not take into account intraoperative changes due to tumor resection, brain shift, and brain deformation [6–9].

*Intraoperative Imaging*. To overcome the limitations of NN based on preoperative imaging, recently it has been proposed to use intraoperative imaging for meningioma surgery: MRI (iMRI), CT (iCT), US (ioUS), and also fluorescent imaging (5-ALA) [10–12].

Soleman et al. have studied if the iMRI could contribute to more extensive surgical resection in complex meningiomas located at the skull base or near eloquent brain areas [11]. In his work, the author presents a series of 27 patients operated on for complex meningioma resection using iMRI; 1 patient died from a fatal postoperative bleeding that was not perceived in iMRI, and 1 patient underwent resection of tumor remnant after iMRI without improvement of the Simpson resection grade. Moreover, the mean duration of the surgical procedure was 449.3 min, with pre- and postresection iMRI mean scan times of 22.5 min and 18.5 min, respectively. The author concluded that iMRI has no relevance on intraoperative approach in meningioma surgery neither for resection grade nor for detection of early postoperative complications.

Uhl et al. have investigated the feasibility of using iCT in brain and spine surgery [12]. In his series, the author describes 34 cases of intracranial meningiomas with a change in surgery in 3 cases in which tumor resection was insufficient. The mean interruption of surgery was 10 to 15 minutes.

Anyhow, both of these techniques cannot be defined as “real-time.” After scan acquisition, the images represent the reality but proceeding with surgery, again, the static intraoperative acquired images became insufficient. Moreover, it is not possible to operate directly under iMRI or iCT control; the images have to be downloaded in the NN system to use a navigated instrument or a pointer. Finally, it is mandatory to consider the cost of an iMRI or an iCT device in money and more importantly in time.

On the other hand, it is necessary to mention the numerous positive aspects of iMRI and iCT [12–15]. Indeed, iMRI provides a detailed multiplanar representation of surgical anatomy on the three canonical orthogonal planes: axial, sagittal, and coronal [13–15]. In routine practice, neurosurgeons are accustomed to these images and this permits a rapid understanding of anatomic structures and targeted lesions. Moreover, iMRI allows acquiring images weighted in different modalities to obtain both anatomical information such as with T1, T2, or FLAIR weighted images and functional information such as with angio-MRI and diffusion weighted and diffusion tensor images.

iCT has achieved a relevant spatial resolution (0.4 to 0.6 mm) that exceeds the majority of iMRI system without the need for major changes of the operating-room work flow. It makes possible to reconstruct the intra-operative acquired volumetric images in order to obtain multiplanar representations, rendering of volume or surface, analysis of perfusion pattern and also to study the vascular district with an angio-iCT scan [12].

Lastly, both iCT and iMRI allow performing a scan of the patient at the end of the surgery. This feature permits assessing the presence of complication shortly after its occurrence and avoiding the necessity for a scan in a second time or a potential second operation [12, 13].

Recently, Cornelius et al., has studied the impact of 5-aminolevulinic acid (5-ALA) in meningioma surgery [10]. His series comprised 19 WHO grade I, 8 grade II, and 4 grade III tumors, in which 94% of the tumors presented positive fluorescence. The author observed that 5-ALA improve the extent of resection in 3/16 of grade I and 6/8 of grade II/III meningiomas but the analysis of the impact of 5-ALA on improving the Simpson grade showed no benefit. Furthermore, although 5-ALA showed residual tumor presence in some cases, further surgical resection was not possible to achieve. Another interesting consideration is that 5-ALA is helpful especially in high-grade meningiomas to visualize tumor tissue infiltrating the parenchyma.

Fluorescent Guided Surgery (FGS) with 5-ALA brings a completely different approach respect to image guided surgery. FGS permits identifying tumor tissue with great specificity but only on the surface of the surgical cavity; to classify an area as 5-ALA positive, it is necessary to expose it in order to evaluate in blue-light. In other words, 5-ALA does not permit obtaining a complete overview of tumor morphology and relationships.

*Intraoperative Ultrasound*. First description of intraoperative application of US in neurosurgery was in 1978 with Reid [16]. Later on, during the 1980s, a lot of neurosurgical applications were reported. Rubin and Dohrmann were the first to recognize that ioUS could be used to localize intracranial masses with great accuracy and to direct surgical resection [17]. Over the years, numerous neurosurgical uses have been described, mainly for localization of brain and spinal cord lesions but also to direct surgical resection or catheter placement [18–22]. Other applications include Doppler studies in vascular malformations, control for aspiration of central nervous system abscess, and evaluation of posterior fossa decompression in Chiari I malformation [23, 24]. IoUS is particularly indicated in neurosurgery because of two specific features that permit to acquire superb images. Mainly the brain's viscoelastic characteristic permits excellent US waves propagation [25]; moreover, the signal is not distorted by interposed tissue like skin and subcutaneous connective.

The major benefit of ioUS is that it is truly real-time [26] and it nowadays reached an excellent temporal and spatial resolution. It shows the real anatomic scenario during all surgery, influencing surgical strategy and, in specific conditions, permitting operating under direct guidance. Another point of value is the great amount of information that is possible to obtain using different ioUS technique, as described below. Moreover, ioUS is relatively cheap if compared with other intraoperative imaging modality like iCT or iMRI. Common US scanners are sufficient to be used in neurosurgery. The only attention regards the probe that has to be specific. Today, the most used are variable band linear probe with operating bandwidth of 11–3 MHz, in order to study both superficial (high frequency) and deeper structures (low frequency) [23].

On the other hand, there are some restrictions linked to ioUS, notably a steep learning curve and operator dependency [24].

The purpose of our study is to review the applications of intraoperative ultrasonography (ioUS) during meningioma surgery, highlighting intraoperative ultrasonographic findings of these lesions. Furthermore we want to emphasize the multiple technical features offered by ioUS and their possible application and impact in meningioma surgery, based on our experience gathered over a 5-year period at our institution and evaluating the current literature in regards.

## 2. Intraoperative Ultrasound in Meningioma Surgery

### 2.1. US Equipment

Last generation standard US portable devices equipped with linear multifrequency (3–11 MHz) probe for deep seated lesion or high frequency (10–22 MHz) for small superficial lesions are usually used.

At our institution, we use a last generation US device (MyLab, Esaote, Italy) with an integrated fusion imaging system that allows for virtual navigation (MedCom GmbH, Germany) with which we are able to perform different surgical steps using one device:

- surgical planning;
- real-time fusion imaging between ioUS and preoperative MRI;
- craniotomy placement;
- transdural lesion evaluation in B-mode;
- recognition of perilesional anatomical landmarks;
- Doppler imaging (echo-color Doppler, power Doppler, and spectral Doppler);
- contrast enhanced ultrasound (CEUS) for vessels recognition and tumor perfusion;
- elastosonography to assess tissue elasticity;
- intraoperative resection control;
- brain shift/deformation correction.

After the craniotomy has been performed, the probe is wrapped in a plastic sterile sheath, coupled with sterile ultrasonic compatible gel. Transdural insonation is started and the surgical field is irrigated with sterile saline solution, in order to avoid air or blood clots between the dura and the transducer. In case of convexity meningioma, the bleeding dura is often coagulated, devascularizing the lesion, partially modifying the findings in regard of the perfusional evaluation with Doppler and CEUS. The lesion is then evaluated on both axis and surrounding structures and standard anatomical landmarks (dural structures, ventricles, choroid plexuses, and arachnoidal folds) are identified. The fusion imaging system, displaying simultaneously real-time ioUS and preoperative MRI, provides an excellent support when interpreting ultrasound imaging and for orientation. Tumor margins and presence of cystic areas or calcifications are evaluated in standard B-mode. Arterial supply, venous drainage, and tumor perfusion are evaluated with different Doppler modalities, as explained below, facilitating the surgical strategy.

A further development in vessels visualization is represented by intraoperative contrast enhanced ultrasound (CEUS), performed injecting intravenously ultrasound contrast agent (UCA) that is made visible by a dedicated algorithm (CnTI). Microbubbles, the size of a red blood cell, are a purely intravascular contrast agent and allow for a real-time intraoperative angiosonography and for a perfusion evaluation.

Further information regarding both tumoral and cerebral tissue characterization is obtained using elastosonograhy, which gives information on the tissue elasticity by associating different chromatic patterns to corresponding tissue elasticity response.

ioUS, an easily repeatable examination, and multiple B-mode scan are performed during tumor debulking, assessing constantly the thickness of the remaining lesion. Virtual navigation, as described in other papers from our group [27, 28], allows also to compensate the brain shift, retraction, and deformation, always maintaining and showing correct orientation, allowing optimal interpretation of ioUS imaging.

After tumor resection has been performed, the cavity is evaluated with navigated B-mode US, and checking eventual residual tumor, evaluating the degree of potential tissue damages and Doppler/CEUS are performed to check vessels integrity.

### 2.2. B-Mode ioUS

**Figure 1:**
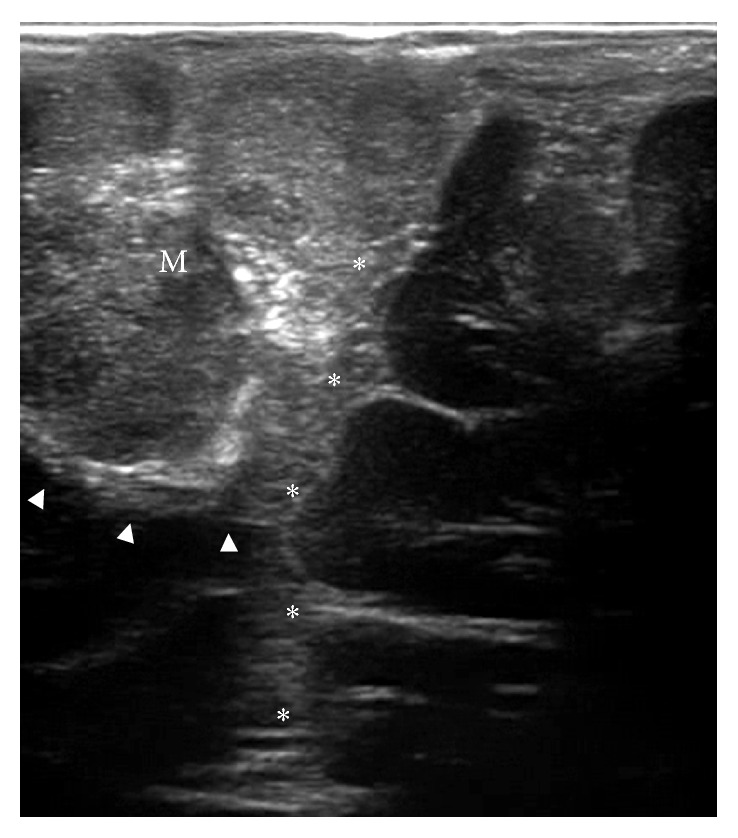
ioUS B-mode scan of a parasagittal meningioma. The lesion (M) appears hyperechoic with a granular aspect and the calcifications are distinctly visible. Tumor/brain interface is recognizable (arrowhead). Relationships with falx cerebri and superior sagittal sinus are evident (asterisks).

The B-mode or brightness mode represents the classical method to acquire an US scan. It is literally an US-tomography, which depicts the section of a structure using a gray scale codification (Figure 1). Every image is constructed converting the intensity of each ecowave reflected from the tissue in a dot on the screen; dot brightness is proportional to the intensity of the ecowave. A B-mode image is evaluated comparing the brightness of the eco of normal tissue to which of tissue in exam. Three situations are possible: hyperechogenicity, hypoechogenicity, and isoechongenicity. Cerebral structures do have different echographic features: choroid plexus, vessels walls, arachnoid, ependyma, skull, dural structures, most tumors and their margins are usually hyperechogenic. Ventricles, cerebrospinal fluid, some tumors are hypoechogenic. White matter, gray matter (tend to be hyperechogenic if compared to white matter), and some tumors appear isoechogenic.

Many authors have described the application of B-mode ioUS in oncological neurosurgery [20, 29–35]. During tumor removal, B-mode ioUS helps in two tasks: (1) tumor identification and (2) resection control. At the beginning of surgery, a B-mode scan permits localizing the lesion and then planning the surgical trajectory avoiding damage to vital structures. During the resection, repeated B-mode scans lead to understanding if remnant tumor tissue is present, where it is, and to estimate its entity [36]. These considerations are mainly true for those tumors that appear hyperechoic by comparison with surrounding brain, for example, metastases, high-grade gliomas, lymphoma, and, in particular, meningiomas [37–39].

**Figure 2:**
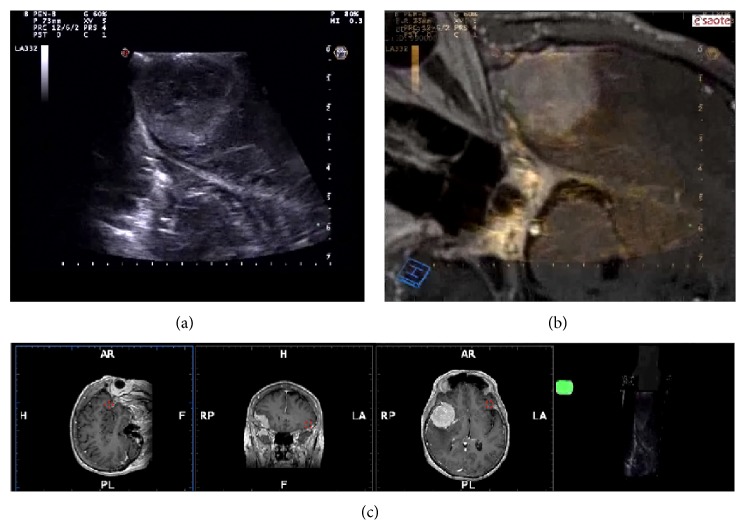
US system screenshot. In the upper panel on the left a standard real-time ioUS B-mode image is displayed; on the right, the corresponding preoperative MRI is fused with real-time ioUS B-mode. In the lower the three standard orthogonal planes (sagittal, coronal, and axial) and the insonation plane panel are displayed.

Meningiomas usually appear hyperechoic, compared to normal brain parenchyma, with a homogeneous pattern and a granular aspect probably due to psammomatous bodies and fine trabeculature (WHO I-II) or with numerous hypoechoic areas of necrotic degeneration (WHO III) [39, 40] (Figures 1, 2, and 4). Calcifications are also observed within the lesion. Tumor margins are usually well depicted even though sometimes edematous brain parenchyma is hyperechoic, and tumor borders may be blurred in case of arachnoidal plane disruption. In some cases, peritumoral vasogenic edema helps in a better delineation of meningiomas boundaries and interfaces by lowering the surrounding brain echogenicity [20].

The lesion is generally explored on the two main axes and measured, and neighbors surrounding structures are examined looking for anatomical landmarks for orientation during surgery. Dural relationships are also taken into account to plan the surgical strategy, especially for lesions in close relation with dural sinuses (Figure 1). After a first morphological evaluation, the lesion is further evaluated with other US modalities as described below. Another notable feature of B-mode is that, being a tomography, a B-mode image permits to study all the tumors margins and relationships also in depth (Figures 1, 2, and 4). Because of the ratio between echogenicity of meningioma and echogenicity of brain parenchyma, during the surgery the tumor tissue remains visible permitting to tackle also the smallest remnant. Multiple B-mode scans are performed during tumor debulking to evaluate the remaining capsule, in order not to trespass it causing damage to surrounding brain parenchyma and to evaluate complete resection, when achievable.

Surely this ioUS modality is not free from negative aspects. At the beginning, it is not user-friendly, in particular because neurosurgeons are accustomed to the three orthogonal planes of MRI and CT (axial, sagittal, and coronal) (Figure 2). Instead, US planes are consequences of probe positioning and this leads to a consistent difficulty in figuring out the spatial orientation of B-mode images. It must be noticed that the steep learning curve of ioUS is related also to the necessity to know the semeiotics of various phenomena that occur during surgery and influence ioUS images. All along meningioma removal it is possible to study the entity of the remnant in order to obtain a complete excision. However, with surgery progression, the surgical cavity became covered by blood clot and the surrounding parenchyma could be damaged by the surgical maneuvers becoming edematous; all these aspects have to be known in order to correctly assess the removal degree [36].

**Figure 3:**
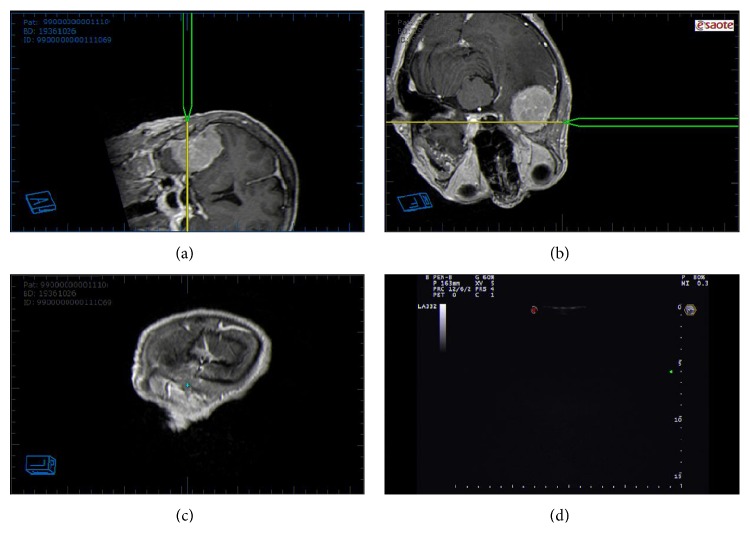
Craniotomy planning. In this screenshot, the pointer position is represented in three reconstructions of preoperative MRI. Using this feature, it is possible to plan the craniotomy site. In the lower-right box is visualized real-time ioUS that does not give any information because of bone shielding.

Another limitation is the B-mode inability to accurately depict tumor relationships with vessels (in particular the smaller) and tumor perfusion pattern. Finally ioUS cannot be used to plan craniotomy because of bone-shielding (Figure 3). For all these reasons B-mode imaging is extremely hard to use and understand alone, in particular when the operator has only a modest experience in ioUS. To overcome this limitation it is helpful to use various ioUS modalities, such as fusion imaging, CEUS, and Doppler. Through the multimodal ioUS study an unexperienced user can better understand each modality. Getting more and more used to ioUS as a qualified sonographer he can obtain numerous additional information from each modality.

On this premises, in our framework, standard B-mode imaging is only the first US applied modality when evaluating the lesion. Fusion imaging with preoperative MRI and the other techniques are described below.

### 2.3. Navigated ioUS

Neuronavigation (NN) is a computational process that associates a real spatial position (in the surgical field) to a virtual spatial position (preoperative imaging study) [41]. Associating this feature to an ioUS scanner, it is possible to fuse the real-time ioUS image to the corresponding reconstructed plane of preoperative MRI in a coplanar fashion [28, 42–46]. In the neurosurgical field, this is extremely relevant for several aspects. Through the use of navigated ioUS probe, it is possible to localize the lesion and to plan the craniotomy going beyond the limitation of US bone shielding [27, 28] (Figure 3). One main limitation of US is the difficulty in spatial orientation that largely is due to the different planes of US images if compared to the traditional orthogonal planes of MRI; moreover, US is not panoramic like MRI. Navigated ioUS displays on the screen the US image with the corresponding MRI; this continuous comparison leads to a better understanding of US image and of its orientation (Figure 2).

**Figure 4:**
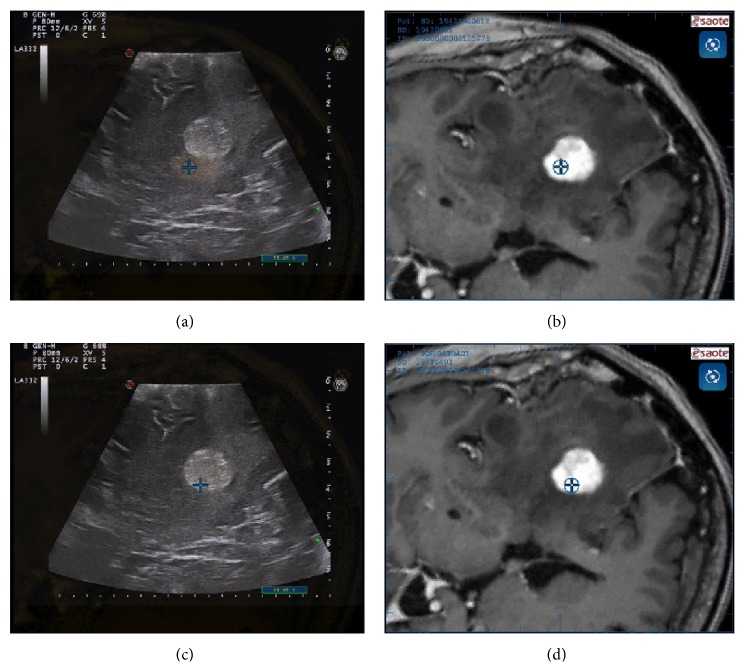
Brain shift correction. Every time a misalignment of ioUS and corresponding preoperative MRI (brain-shift) is appreciable, it is possible to realign the images in order to regain the system accuracy. In the upper panel, a misalignment in visible trough the fusion imaging. In the lower panel, the shift is fixed. In the right boxes, preoperative MRI are displayed.

As stated before, NN system suffers from brain-shift and tissue distortion. Through the use of a Navigated ioUS system, it is possible to correct these errors several time during surgery, always obtaining the best accuracy possible [28] (Figure 4).

In meningioma surgery, all these positive aspects of navigated ioUS are extremely attractive. In complex meningiomas, which have close relationships with vessels or other vital structures, the comparison with preoperative MRI permits to better understand the surgical anatomy, avoiding unintentional damages [38]. Regarding brain-shift phenomena, it was demonstrated that meningioma surgery causes the highest level of brain deformation/shift leading to a premature loss of accuracy of the neuronavigation system [6]. In this setting, the possibility to correct brain-shift for all the surgery is particularly relevant.

In matter of limitations of navigated ioUS, it must be considered that the enrichment of information obtainable by comparison with MRI is based on a preoperative acquired image. For this reason, it is impossible to represent the real situation of surgical field. Moreover, it is impossible to perform biological study as quantification of flow entity and direction in a vessel or to obtain information about tumor perfusion, stiffness, or changed relationships.

In our experience, fusion imaging proved to be accurate [27, 28]; it allows to correctly place the craniotomy and provides better image interpretation and orientation (Figures 2 and 3). Brain shift/deformation correction is performed on a routine basis to maintain the proper alignment for better orientation and understanding of US imaging (Figure 4). It can also be coupled with each of the US modality described in the paper.

### 2.4. Doppler ioUS

The Doppler effect or Doppler shift is a physics phenomenon consisting in change of frequency and wavelength of a mechanical wave that is reflected from a moving object. Doppler US through a Fourier transform evaluates the change in frequency of a US wave when it is reflected from flowing blood in order to reconstruct an image. In general, Doppler imaging can be used to study three aspects of a vessel: presence or absence of flow and direction and velocity of flow. The principal limitation is the dependency from the angle of insonation; if the angle approaches 90° (probe perpendicular to the vessel), Doppler signal is significantly decreased until it disappears, vice versa the signal increases when the plane of insonation is parallel to the vessel [47].

Three main techniques exist for Doppler imaging: Color Doppler, spectral Doppler, and Power Doppler.

**Figure 5:**
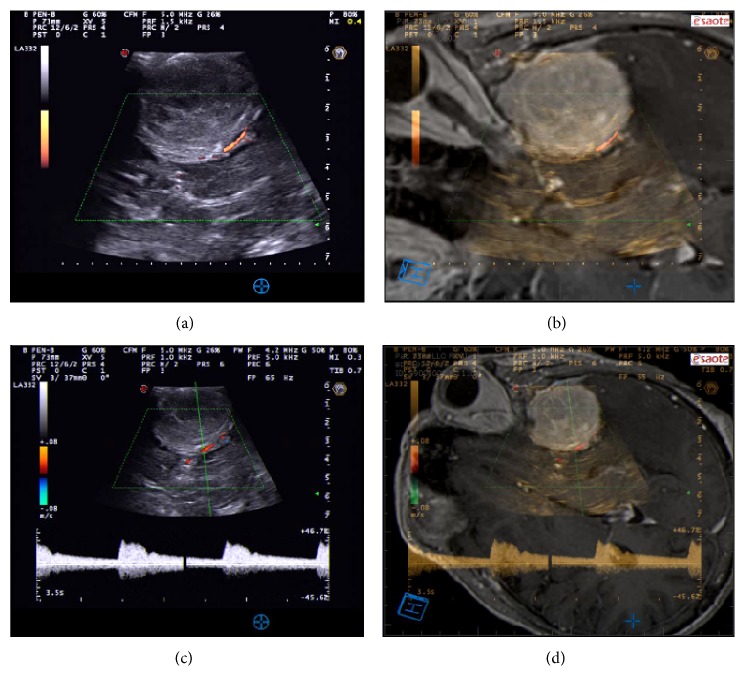
Doppler imaging. In the upper panel, power Doppler scan depicts the relationship between tumor and middle cerebral artery; this technique is less sensible to insonation angle but cannot represent flow direction and velocity. In the lower panel, color Doppler and spectral Doppler bring information about flow direction and velocity with quantification of the velocity through spectral Doppler. In the right boxes, fusion imaging between ioUS Doppler scan and corresponding preoperative MRI is displayed.

Color Doppler allows identifying the presence and the direction of flow in vessels in a B-mode image, in which the operator place a color box, that correspond to the region scanned to acquire Doppler signal. In the color box, it is possible to observe flow direction and velocity trough an encoded color scale. Conventionally, blue indicates flow away from the probe and red flow towards the probe [47] (Figure 5).

Power Doppler or Doppler angiography is a technique that represents on a B-mode image only the magnitude of Doppler signal rather than velocity and direction. In other words, it displays the amplitude of red blood cells present in an area. Power Doppler uses a single color scale in which the increase in brightness corresponds to an increase in signal strength [47] (Figure 5).

Spectral Doppler is usually combined with B-mode and color Doppler technique and permits evaluating flow velocity in the sample volume (selected area in B-mode image). Usually, after having set the volume sample, B-mode and color Doppler are frozen, in order to improve frame rate analysis, achieving a more precise measurement. Spectral Doppler produces an analysis graph, with time on horizontal axis and velocity on vertical axis. The brightness of the spectral trace represents the backscattered power of Doppler signal at each velocity [47] (Figure 5).

Each of these Doppler techniques has proper advantages and limitations.

Color Doppler gives an overview about presence of flow and shows flow direction in a selected region. On the other hand, it suffers from angle dependency, it is subject to aliasing and has low temporal resolution because of low frame rate due to the necessity of several scan to obtain a reliable estimation of flow velocity [47].

Power Doppler is more sensitive to low flow vessels permitting to study tumor perfusion; moreover, it is not subject to angle dependency and does not need a sampling technique. Its main limitations are the impossibility to show flow direction and velocity, and it suffers from very poor temporal resolution because it needs a high degree of frame averaging and for this reason it is very sensitive to probe motion [47].

Spectral Doppler has an exceptional temporal resolution giving a precise estimation of flow during all the cardiac cycle but it is angle dependent and does not give anatomical information [47].

Numerous studies have investigated the utility of intraoperative Doppler imaging for vascular and neoplastic lesions in neurosurgery [38, 48–54].

In particular, Solheim, in 2009, studied the application of power Doppler in meningioma surgery [38]. The author concludes that in most cases power Doppler could be useful in visualizing feeding arteries and neighbors vital vessels leading to a rapid and safe intracapsular tumor resection minimizing the risk of damage to important vascular structures. Anyway, he underlines that this technique is limited by the difficulty to study low-flow vessels and by the blooming artifact that tends to overestimate the smaller vessels.

Otsuki, in 2001, described one case of petroclivotentorial meningioma studied with various ioUS technique among which color Doppler [55]. He emphasizes the limitation of blooming artifacts that make Doppler signal to overwrite vessel walls bringing incorrect information.

Our findings are consistent with those from the literature (Figure 5).

### 2.5. Contrast Enhanced ioUS

Ultrasound contrast agents (UCA) are purely intravascular contrast agents, generally used in to evaluate organ or lesion perfusion and vessel anatomy [56]. In 2000 the initial studies regarding the use of first generation UCA in liver US were published [56, 57]. Two years later, sulphur hexafluoride (SonoVue, Bracco, Milan) introduced the concept of real-time low mechanical index (MI) contrast enhanced US (CEUS), allowing for a continuous imaging [58].

Three types of UCA are approved in Europe today:

- Levovist (air with a galactose/palmitic acid surfactant) (Schering, introduced in 1996),
- Optison (octafluoropropane with an albumin shell) (Amersham, introduced in 1998),
- SonoVue (sulfur hexafluoride with a phospholipid shell) (Bracco, introduced in 2001).

In general UCA has a microbubble (MB) structure (gas stabilized by a shell) and behaves as a purely intravascular agent. For this reason, UCAs are used to visualize blood flow and vasculature tree in a structure/organ through enhancement of blood echogenicity. Study of MB distribution requires a specific imaging technique in order to suppress linear tissue US signal visualizing only the nonlinear harmonic echo of MB [59–61].

There are two mechanisms to obtain the nonlinear response of MB: through MB oscillations in low acoustic pressure (minimizing disruption), and high energy nonlinear response from MB disruption with high acoustic pressure [62].

First generation UCAs like Levovist require high Mechanical Index (MI) US leading to MB disruption and limiting US frame-rate in order to permit refill of MB into vasculature.

Second generation UCAs like SonoVue are more stable permitting to acquire nonlinear signal at low MI. This leads to minimal MB disruption and therefore a continuous study of structure/organ for several minutes, dynamically evaluating the enhancement in real-time.

Over the years, an incredible number of papers have studied the UCA application in liver and many other organs.

Concerning intraoperative setting in neurosurgery, few studies have been published [40, 63–67].

Kanno et al. obtained intraoperative tumors visualization in 40 cases through the use of a first generation UCA and therefore he obtained only discontinue low frame-rate images [66].

Engelhardt et al. published 7 cases of glioblastoma in which a second generation UCA allowed to perform also time-intensity curves thanks to continuous imaging [63].

Hölscher et al. has described the phase inversion harmonic imaging technique using Optison in 13 patients (8 middle cerebral artery aneurysms, 5 arteriovenous malformation) [65]. The author concluded that CEUS through phase inversion harmonic imaging enables intraoperative visualization and anatomical study of vascular pathologies and that the flow dynamics of these lesions can be displayed in real-time allowing to evaluate the success of a clipping procedure.

He et al. used a second generation UCA in 29 cases (22 gliomas and 7 meningiomas) concluding that intraoperative CEUS is useful in locating the lesion, in defining the border between the tumor and healthy brain and in detecting residual tumor [64].

Our group recently published two studies concerning intraoperative CEUS safety and its application in tumor evaluation and removal [40, 67]. We have observed that intraoperative CEUS with SonoVue is a valuable real-time tool to obtain anatomical and functional information such as vascularization and tissue perfusion pattern [40]. In case of gliomas surgery using CEUS it is possible to differentiate between low-grade and high-grade tumors and in particular cases to find anaplastic areas within otherwise considered low-grade lesion [67].

**Figure 6:**
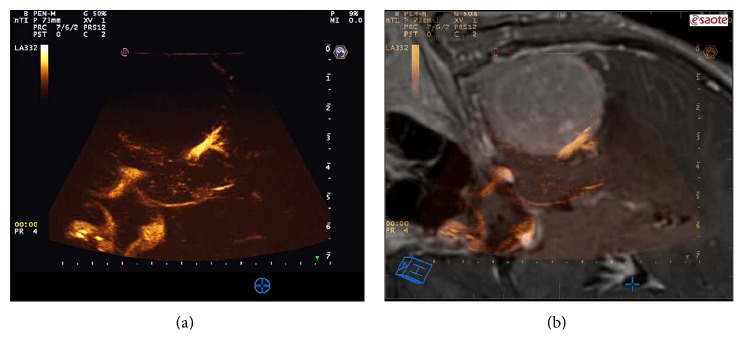
CEUS imaging. In the left box, CEUS scan of the lesion is displayed; in the right box, fusion imaging between intraoperative CEUS and corresponding preoperative MRI aid in US interpretation. Because the main vascular supply of this lesion was from dural attachment, once it was coagulated, no perfusion of the lesion was noticeable. The principal vascular structures are clearly visible: plexus of Willis, basilar tip, cavernous sinus, and the neighbor middle cerebral artery.

**Figure 7:**
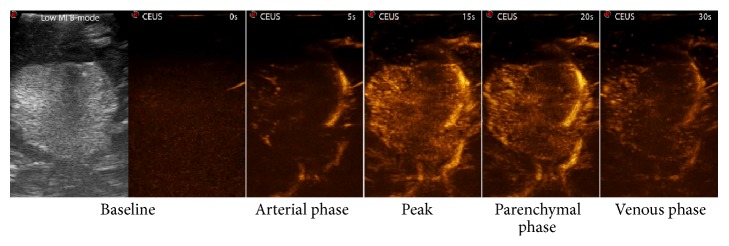
CEUS phases. In this picture, a low mechanical index (MI) B-mode scan is depicted together with screenshot of the main phases of contrast enhancement dynamics. In the arterial phase, the main feeders are clearly visible. In peak and parenchymal phase, it is possible to differentiate hyper- or hypovascularized areas within the tumor. In the venous phase, multiple small draining vessels are recognizable.

Performing CEUS in meningioma surgery, we obtained useful information regarding their perfusion prior to resection (Figures 6 and 7), identifying a typical pattern: meningioma shows an intense and rapid contrast enhancement (due to a very fast arterial phase) with higher degree of contrast enhancement and faster peaks in higher grades. Generally, contrast enhancement is centripetal having the major supply from the dural attachment and surrounding vessels (Figures 6 and 7). The slow venous drainage is not always visible. Intratumoral major vessels are visible only in higher grades meningiomas, which present some hypoechogenic/necrotic areas. Tumor borders are distinctly visible in all cases [40] (Figure 7).

The great benefit of intraoperative CEUS in meningioma surgery is the possibility to visualize the vessels surrounding the tumor, not only on the surface of the surgical cavity, as achieved with white light microscopy and FGS [10], but being CEUS a tomographic image, also in the depth (Figures 6 and 7). This permits to accurately evaluate and identify arterial feeders and surrounding vessels, allowing to carefully plan the surgical strategy. It is possible in fact to precisely target arterial feeders obtaining a complete tumor devascularization. After having coagulated the dural attachment CEUS might be repeated to evaluate if the tumor is completely devascularized or if there are other suppliers to be closed (Figure 7). Furthermore, CEUS allows the identification of large surrounding vessels: coupling CEUS with virtual navigation permits to localize these vessels in the three dimensional frame within the surgical field, allowing for a safer dissection of these vital structures (Figure 6).

### 2.6. Intraoperative Elastosonography

Elastosonography is a noninvasive representation of a specific mechanical characteristic of a tissue: the elasticity, that is, the property of a tissue to deform under a given forces and then to restore to its original shape after distortion. Elasticity is commonly defined by the amount of deformation (strain) resulting from a given stress. Elasticity evaluation is obtained studying the deformation of a tissue in response to the application of an external or internal force [68].

Today, many different elastosonographic techniques exist; they are classified in accordance to which type of force they use to obtain a deformation in the tissue [68]. The stimulation force could be either dynamic (mechanical or US induced) or fluctuating so slowly that it is named “quasi-static” (mechanical induced). In the last case the stimulation force can be either an active external displacement of tissue or a passive internal displacement physiologically induced [68]. Whatever the stimulation technique is, all different elastographic methods aim to show the shear elastic modulus of the examined tissue.

Actually the most used technique is the quasistatic strain elastosonography (SE), which aims to display strain properties of a tissue in qualitative terms. SE is figured in real-time, coding information related to tissue strain, since regions of different stiffness react differently to force stress (ultrasound probe compression and release or due to physiological tissue motion linked to vascular pulsation) [68]. An object, subject to stress, distorts proportionally to the intensity of the applied stress and depending on the material it is made of; it is possible to evaluate the modification of the echo signal and thus to compute how the different tissues distort (if they are soft) or move (if they are hard) compared to the probe position. The representation of tissue elasticity is obtained associating different chromatic patterns to different tissue elasticity response.

There are only few reports in oncological neurosurgery about the elastographic implementation of ioUS [69–73].

Scholz et al., in 2005, described the use of an US based real-time strain imaging method to study the elastic properties of brain neoplasms [69]. In his series there are various tumors and one case of atypical meningioma. He observed that some tumor exhibits the same stiffness of normal tissue, other lower or higher; the meningioma case presented higher strain than brain parenchyma. The conclusions were that US based real-time strain imaging is feasible, safe and offers information regarding the tumor.

In 2009 Uff et al. presented for the first time elastosonographic acquisition obtained through arterial pulsations during spinal cord surgery [73]. The results highlighted that strain data correlate with the surgeon's finding of stiffness of the tissues, and areas of higher stiffness at tumors boundaries were found to be related to the cleavage planes.

Selbekk et al., in 2005, 2010, and 2012, investigated the utility and feasibility of a strain imaging method to discriminate between tumor (low grade gliomas and metastasis) and normal brain [70–72]. He observed that tumor areas are characterized with lower strain levels than those of healthy tissues and that tumor interpretation could be different on the two modalities. Another conclusion was that strain imaging leads to better discrimination between glial tumor and normal tissue if compared to standard B-mode. Under technical aspects, he found that the brain motion due to arterial pulsation is sufficient to generate an elastogram.

**Figure 8:**
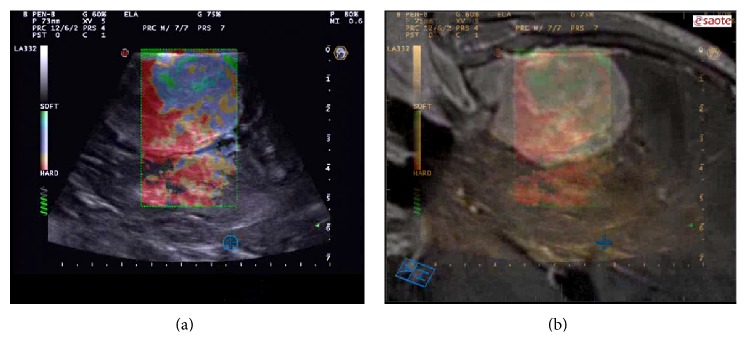
Elastosonography imaging. In the left box is visualized the elastosonogram (strain elastography) of the lesion; in the right box, it is fused with corresponding preoperative MRI. Elastosonography shows that the tumor has two different consistencies; this finding was confirmed by surgeon feelings. Deeper into the lesion, temporal lobe has higher stiffness if compared with normal findings; this is due to mechanical compression from meningioma.

In our multimodal ioUS study for meningioma removal, SE is considered in order to obtain a virtual palpation of the tumor. SE is performed before opening the dura mater, relying only on physiological tissue movement due to vascular pulsation, in order to avoid cortical damages. As partially stated by Uff [73], we found that SE provides information about meningioma consistency and homogeneity of stiffness (Figure 8). These notions are extremely relevant in order to know what to expect during surgical removal and to probably better evaluate and identify tumor/brain interface.

## 3. Conclusions

ioUS is definitely a valuable tool in meningioma surgery as already stated for other brain neoplasm. It ensures a rapid, repeatable, and cost effective real-time intraoperative imaging.

Standard B-mode US offers significantly useful morphologic information, which can be further implemented with fusion imaging for better US imaging understanding and orientation. The integration with different Doppler modalities as well as CEUS offers incomparable information regarding tumor vascularization and perfusion, thus facilitating the surgical strategy. Elastosonography seems to be a promising tool especially to evaluate tumor borders, eventual parenchymal infiltration, and tumor consistency.

However, US in neurosurgery is yet not a widespread technique: US is a quite complex investigation and is highly operator dependent. Furthermore basic neurosurgical semeiotics needs surely to be implemented and specific training on US physics and “knobology” is required [36].

Our approach when using ioUS in meningioma surgery is not to use this tool alone to achieve complete resection, rather to explore its various possibilities and to obtain as much information as possible to achieve a safer and more complete resection.

Of course ioUS cannot provide the surgeon with all needed information and it has to be integrated with other imaging modalities, when available, and surgical tools to plan the best surgical strategy and to offer the best procedure.

Further studies are warranted to fully investigate US role in neurosurgery, with a particular attention to recent US techniques such as CEUS an elastosonography. However, the multimodal US imaging approach in meningioma surgery seems to offer a vast array of information, yet to be fully understood, that seems able to facilitate the surgical strategy.

## Abbreviations

5-ALA: 5-AminoLevulinic acid
B-mode: Brightness mode
CEUS: Contrast enhanced ultrasound
FGS: Fluorescent Guided Surgery
iCT: Intraoperative computed tomography
iMRI: Intraoperative magnetic resonance imaging
ioUS: Intraoperative ultrasound
MI: Mechanical index
MB: Microbubbles
NN: Neuronavigation system
SE: Strain elastography
UCA: Ultrasound contrast agent
US: Ultrasound.
